# Recent Advances of Small Extracellular Vesicles for the Regulation and Function of Cancer-Associated Fibroblasts

**DOI:** 10.3390/ijms252312548

**Published:** 2024-11-22

**Authors:** Chengdong Liang, Maoye Wang, Yongli Huang, Judy Wai Ping Yam, Xu Zhang, Xiaoxin Zhang

**Affiliations:** 1 Jiangsu Key Laboratory of Medical Science and Laboratory Medicine, School of Medicine, Jiangsu University, Zhenjiang 212013, China; lcddcl0920@163.com (C.L.); wmyujs@foxmail.com (M.W.); 2212313064@stmail.ujs.edu.cn (Y.H.); 2 Department of Pathology, Li Ka Shing Faculty of Medicine, The University of Hong Kong, Hong Kong 999077, China; judyyam@pathology.hku.hk

**Keywords:** small extracellular vesicles, cancer-associated fibroblasts, crosstalk

## Abstract

Cancer-associated fibroblasts (CAFs) are a heterogeneous cell population in the tumor microenvironment (TME) that critically affect cancer progression. Small extracellular vesicles (sEVs) act as information messengers by transmitting a wide spectrum of biological molecules, including proteins, nucleic acids, and metabolites, from donor cells to recipient cells. Previous studies have demonstrated that CAFs play important roles in tumor progression by regulating tumor cell proliferation, metastasis, therapeutic resistance, and metabolism via sEVs. In turn, tumor-derived sEVs can also regulate the activation and phenotype switch of CAFs. The dynamic crosstalk between CAFs and cancer cells via sEVs could ultimately determine cancer progression. In this review, we summarized the recent advance of the biological roles and underlying mechanisms of sEVs in mediating CAF-tumor cell interaction and its impact on cancer progression. We also reviewed the clinical applications of tumor- and CAF-derived sEVs, which could identify novel potential targets and biomarkers for cancer diagnosis, therapy, and prognosis.

## 1. Introduction

The tumor microenvironment (TME) comprises a highly organized ecosystem consisting of cancer cells surrounded by diverse non-malignant cell populations collectively immersed in a modified extracellular matrix characterized by vascularization [1]. Cancer-associated fibroblasts (CAFs), key cellular components of the TME, play a vital role in facilitating cancer progression and metastasis [2]. CAFs are defined as a group of cells that synthesize collagen in connective tissue and express mesenchymal markers, such as αSMA, FAP, and Vimentin, but lack the expression of epithelial and endothelial markers [3,4]. CAFs can derive from various cell types within the TME, encompassing not only normal resident fibroblasts, but also stellate cells, mesenchymal stem cells (MSCs), adipocytes, pericytes, and additional stromal cells [5,6,7,8,9,10,11]. The varied origins of CAFs give rise to functionally and phenotypically heterogeneous populations within the TME. This diversity shows the dynamic nature of CAFs, transitioning into distinct subtypes across different TME contexts, which reflects their pleiotropic roles and functional heterogeneity. Consequently, the mechanisms through which CAFs modulate cancer progression are not only diverse, but also highly dependent on the tissue context [1]. Therefore, it is imperative to delve deeper into the communication mechanisms between CAFs and tumor cells to develop and implement precise and effective treatment strategies.

Extracellular vesicles (EVs) are a heterogeneous group of vesicles delimited by lipid bilayers. Small extracellular vesicles (sEVs), often referred to as a subset of extracellular vesicles (EVs) with diameters less than 200 nm, are secreted by various stromal cells within the TME [12,13]. Research has revealed that sEVs encapsulate a wide array of bioactive cargoes, including nucleic acids, proteins, lipids, and metabolites, and these cargoes can be transferred to recipient cells, significantly impacting their biological functions [14,15]. sEVs play a pivotal role in intercellular communication, exerting both stimulative and inhibitory effects on cancer progression and therapeutic response. However, further investigations are warranted to elucidate the biological roles and molecular mechanisms of sEVs released by distinct cellular subsets within the TME. Of note, many previous studies have named EVs with sizes ranging from 40 to 160 nm as ‘exosomes’. However, according to MISEV2023, most of the abovementioned ‘exosome’ research refers to a broad population of EVs and not to EVs that are released via specific biogenesis pathways [16]. Therefore, the most recent guidelines from ISEV discourage the use of ‘exosome’, unless the subcellular origin can be demonstrated [16]. In this review, we use the universal term sEVs, which is recommended by the latest MISEV guidelines, to name the terms involving exosomes in existing studies.

Currently, accumulative evidence suggests that there is a dynamic transfer process of sEVs between cancer cells and CAFs in the TME [17]. Cancer cell-derived sEVs can induce the activation and switch of phenotypes of CAFs, and in turn, CAF-derived sEVs play pivotal roles in regulating cancer progression, therapeutic resistance, and metabolic reprogramming [14,18,19,20,21]. Utilizing these sEVs and their cargo as cancer biomarkers and therapeutic delivery vehicles represents a promising strategy for cancer diagnosis and treatment. In this review, we discussed the current insights into sEVs’ role in facilitating cell–cell communication between CAFs and cancer cells and assessed their cargo’s potential as therapeutic agents, diagnostic biomarkers, and prognostic predictors in cancer.

## 2. Cancer-Associated Fibroblasts (CAFs)

The tumor microenvironment (TME) refers to the surrounding microenvironment in which tumor cells exist, and the proposal of the ‘seed and soil’ doctrine has revealed the critical role of the TME in cancer progression [22]. Indeed, the interaction between tumor stroma and malignant cells has the ability to remodel the extracellular matrix (ECM), which can regulate cancer proliferation, metastasis, therapeutic resistance, immune response, etc. [23]. Cancer-associated fibroblasts (CAFs) are an indispensable component of the TME, as they secrete various cytokines, growth factors, and sEVs that actively promote tumor malignant progression, including proliferation, metastasis, angiogenesis, and chemoresistance [24]. Further insights into the origin, subtypes, and functions of CAFs, which are elaborated upon below, may contribute to a deeper understanding of the role of CAFs in cancer.

### 2.1. Origin of CAFs

Mounting evidence shows that CAFs constitute not merely a singular cell type, but a heterogeneous cell population [25]. The functions and characteristics of distinct CAF subtypes are primarily influenced by their originating organs and cells, as well as the diverse TME in which they are situated. Although well-developed lineage-tracing and single-cell analysis studies have revealed multiple sources of CAFs in different tumor cell lines, their activation and transformation mechanisms need to be further explored and investigated [6,26,27,28].

First, CAFs can originate from normal resident fibroblasts, which are activated by cytokines and other factors secreted by tumor cells. For instance, bladder cancer cells secrete exosomes enriched with TGF-β, leading to the activation of SMAD-dependent signaling in exosome-induced CAFs, subsequently prompting the differentiation of fibroblasts into CAFs [10]. In addition, Nodal derived from melanoma and colorectal cancer (CRC) tissue converts normal fibroblasts into CAFs, while Snail and TGF-β signaling pathways are activated in fibroblasts [29]. As a member of the serine protease family, HTRA1 promotes the transformation of normal fibroblasts to CAFs in gastric cancer by activating NF-κB signaling to upregulate the expression of bFGF/FGF2 [30]. In summary, inhibiting the transformation of normal resident fibroblasts into CAFs may hinder tumor malignancy progression. This inhibition can be achieved by either downregulating proteins that facilitate CAF differentiation or blocking the exosome-mediated communication essential for their transformation. Therefore, therapeutic strategies targeting CAFs hold the potential to diminish their tumor-promoting activities.

MSCs (mesenchymal stem cells) in the TME are an additional major source of CAFs. Recent evidence has shown that TGF-β secreted by leukemic cells when bone marrow mesenchymal stem cells (BM-MSCs) are co-cultured with leukemic cells can activate the SDF-1/CXCR4 pathway to trigger the conversion of MSCs to CAFs [31]. Notably, a recent study constructed a human cell-derived stroma-rich pancreatic cancer model, which revealed the origin of CAFs from transplanted human adipose MSCs by single-cell-based RNA sequencing and histological analysis [32].

Stellate cells of MSC origin, confirmed by cell lineage tracing [33], which are also a source of CAFs, particularly myofibroblasts, are crucial for promoting tumor metastasis. For instance, liver stiffness due to the presence of tumors activates the conversion of hepatic stellate cells (HSCs) into myofibroblasts. Mechanistically, a transcription-regulated histone acetyltransferase E1A-binding protein, p300, enhances the expression of regulatory transdifferentiation genes in HSCs [34]. In addition, hepatocellular carcinoma cell-derived exosome miR-21 can target PTEN and facilitate the activation of the PDK1/Akt pathway in HSCs cells, thereby converting to CAFs and further contributing to malignant tumor progression [9], suggesting that a communication pathway mediated by sEVs between hepatocellular carcinoma (HCC) cells and HSCs exists and that inhibiting this pathway may represent a promising therapeutic measure to reduce the source of CAFs and confer anti-tumor effects in HCC.

**Figure 1:**
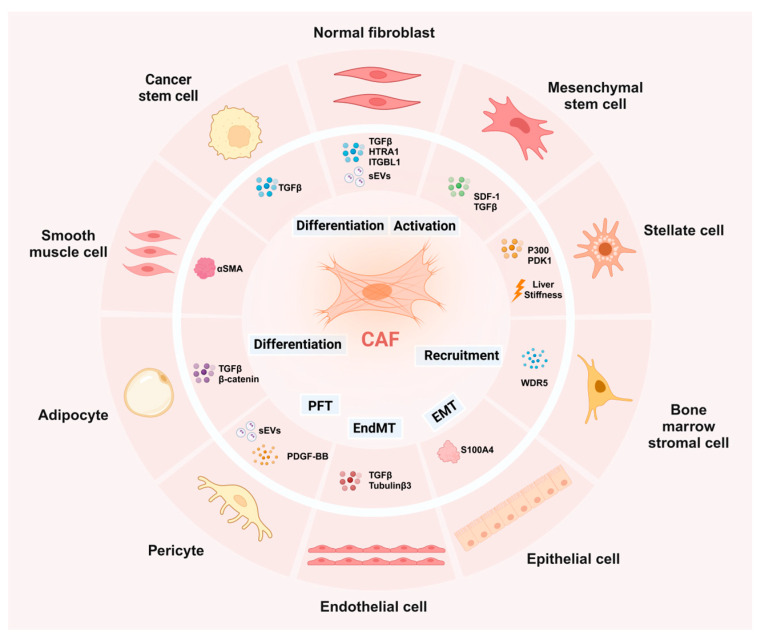
The origins of CAFs. CAFs are mainly derived from normal fibroblasts, MSCs, stellate cells, bone marrow stromal cells, epithelial cells, endothelial cells, pericytes, adipocytes, tumor stem cells, and smooth muscle cells.

Moreover, some cells with non-fibroblast lineages can also transdifferentiate to CAFs under certain stimuli, for example, epithelial cells via EMT [35,36], endothelial cells via EndMT [37,38], pericytes [39,40], bone marrow cells [41], adipocytes [42,43], and tumor stem cells (CSCs) [44] (Figure 1). The extensive number of mechanistic studies addressing the origin of CAFs to date has greatly enriched our understanding of the heterogeneity of CAFs, as the major stromal cells in the TME, play an essential role in tumor proliferation, metastasis, and drug resistance [45]. Targeting the origin of CAFs is anticipated to be a promising strategy with developmental potential in cancer therapy.

### 2.2. Biomarkers, Subpopulations, and Heterogeneity of CAFs

CAFs display significant plasticity and consist of various functionally distinct subtypes [1]. Recently, a key study exploiting spatially resolved single-cell imaging mass cytometry (IMC) to analyze CAFs has shed light on the relationship between CAF phenotypes and patient prognosis in NSCLC [46]. Due to the heterogeneity of phenotypes, biomarkers, and function of CAFs in diverse tumor types, it is difficult to determine their various subtypes. The characterization of CAF heterogeneity is critical for the advancement of potentially effective approaches for cancer treatment [47].

In the past decade, diverse markers have been suggested to define CAFs, such as α-SMA, FSP-1, FAP, Vimentin, PDGFR-α, and PDGFR-β. Although none of these biomarkers is uniquely expressed by CAFs, a composite profile of these markers can still delineate CAFs effectively. α-SMA, also known as ACTA2, is a classical CAF marker to identify CAFs with a myofibroblast morphology, which involves cell motility, structure, and integrity [48,49]. By contrast, one illuminating case illustrated that α-SMA expression drops significantly in the stroma of pancreatic and prostate cancers, which also emphasized the heterogeneity of CAFs [50]. Additionally, α-SMA-positive fibroblasts are highly associated with a higher risk of recurrence and lower overall survival in colon cancer patients [51]. However, α-SMA is also highly expressed by other cell types, such as fibroblast reticular cells, smooth muscle cells, lymphocytes, and pericytes of blood vessels [52]. The serine protease fibroblast activation protein (FAP) represents a characteristic marker for CAFs across a spectrum of tumors and is pivotal in ECM remodeling, angiogenesis, and chemoresistance. It emerges as a promising therapeutic target for inhibiting CAFs’ activity [53,54]. Nonetheless, the FAP is also not exclusively expressed by CAFs, as it is highly present in other stromal mesenchymal cells [55]. CAF marker fibroblast-specific protein 1 (FSP1), also known as S100 calcium-binding protein A4 (S100A4), is involved in the prevention of carcinoma through encapsulation of carcinogens and collagen production [56]. Vimentin is an intermediate filament protein and another CAF marker, which is related to motility [57] and the structural integrity [58] of the cell. Vimentin-expressing CAFs can promote metastasis in lung adenocarcinoma by interacting with heterotypic tumor cells [59]. Another study reported that Vimentin-positive CAFs with a negative expression of α-SMA were relevant to poor survival in PDAC [60]. Both PDGFR α and β are employed as general markers for CAFs as well, which are tyrosine kinase receptors located on the surface of fibroblasts. Although no PDFGRs shows significant expression in CAFs, they can be expressed more stably in fibroblasts and be applied as more general markers in combination with more specific CAF markers [61].

At present, researchers have determined various subtypes of CAFs in multiple types of tumors mainly based on the expression of the abovementioned biomarkers, functions, and phenotypes in different CAFs. By epigenomic, transcriptomic, and proteomic profiling, researchers have divided the CAF subpopulation into three functional categories across multiple solid tumor types and species, including steady state-like (SSL) CAFs, mechanoresponsive (MR) CAFs, and immunomodulatory (IM) CAFs. This study emphasized a ‘push–pull’ dynamic among CAF subpopulations under the selective disruption of an underlying mechanical force or immune checkpoint inhibition therapy, the balance of which provided translational implications [62].

In pancreatic ductal adenocarcinoma (PDAC), Öhlund et al. identified two spatially separated and mutually exclusive subtypes of CAFs [50]. Myofibroblastic CAFs (myCAFs), which are contractile and perform stroma remodeling, are located adjacent to tumor cells and highly expressed in α-SMA. While inflammatory CAFs (iCAFs) are defined by low α-SMA expression and are more distantly distributed throughout cancer cells, and they can foster the growth and proliferation of neoplastic cells by secreting a variety of inflammatory cytokines and chemokines, such as IL-6, IL-11, and CXCL12, in pancreatic tumors [50]. In addition, Elyada et al. uncovered a third subtype of CAFs that expressed MHC II molecules and CD74 by single-cell RNA-seq analysis and has the capacity to modulate immunity, termed antigen-presenting CAFs (apCAFs) [63]. Recently, using single-cell RNA sequencing, a key study described a new subtype of CAFs, named meCAFs, which had a highly activated metabolic state in PDAC, and patients with ample meCAFs had a higher risk of metastasis and a poor prognosis. However, a better response to immunotherapy still existed in the same patients with PDAC [64]. It is noteworthy that the different subtypes of CAFs are dynamic and can rapidly be reverted to multiple phenotypes based on their tumor microenvironment [63].

In a genetically engineered mouse model of breast cancer, four distinct subtypes of CAFs were defined by single-cell RNA sequencing. Matrix CAFs (mCAFs) were significantly enriched for gene sets associated with the ECM and EMT. Gene sets detected for vascular CAFs (vCAFs) were connected to vascular development and angiogenesis. Cycling CAFs (cCAFs), which were the proliferating segment of CAFs, had a strong expression of cell cycle-related gene sets. At last, differentiation-related genes dominated in developmental CAFs (dCAFs) [65]. Additionally, using flow cytometry, immunohistochemistry, and RNA sequencing, researchers demonstrated four subtypes of CAFs, which are named CAF S1-S4 in breast cancer. CAF S1 and CAF S4, accumulating in the lymph nodes, were myofibroblastic phenotypes and involved in cancer cell invasion. CAF S1 generated EMT transition and promoted cancer cell migration through TFG-β and CXCL12 pathways. CAF S4 was highly contractile and induced cancer cell invasion through NOTCH signaling [66,67]. Of note, a key study uncovered two special surface markers, CD10 and GPR77, to define a novel CAF subset associated with chemoresistance and poor survival in breast and lung cancer patients. More importantly, this study suggested a promising therapeutic strategy by isolating and targeting CD10 + GPR77 + CAFs for CSC-driven solid tumors [45].

Like immune cells, CAFs comprise diverse cell subpopulations that respond to various stromal stimuli, exhibit distinct secretory phenotypes, and perform unique biological functions within the dynamic tumor microenvironment. Numerous contemporary studies have defined different CAF subpopulations based on their cellular origin and surface markers. However, the inherent heterogeneity of CAFs has led to many conflicting reports. Consequently, to precisely and specifically define CAF subpopulations, it is necessary to comprehensively explore additional CAF characteristics, such as activation stages and spatial distributions, in future studies.

## 3. Extracellular Vesicles (EVs)

Extracellular vesicles (EVs) are a heterogeneous group of particles encapsulated by a lipid bilayer and released by all cell types [16]. In 1967, Wolf P. discovered a kind of minute particulate material in human plasma by ultracentrifugation, which was rich in phospholipid and showed coagulant properties resembling those of Platelet Factor 3 [68]. In a pivotal study in 1981, EVs were understood to serve as conduits for removing metabolic waste from the intracellular to the extracellular [69]. However, Ratajczak, J. et al. found that there are potential bioactive components, such as protein and mRNA, in membrane-derived vesicles (MVs) that significantly promote pluripotency in mouse hematopoietic stem cells, suggesting the importance of EVs as a medium for intercellular communication [70]. Importantly, accumulating evidence shows that EVs play a pivotal role in a variety of malignant progression processes, including tumorigenesis, proliferation, metastasis, angiogenesis, drug resistance, and stromal interactions [71,72]. It is noteworthy that these mechanisms have primarily been identified through studies on small extracellular vesicles (sEVs) [17,73,74,75]. Interestingly, the intercellular transfer of sEVs from cancer cells to stromal cells has been discerned as a pivotal mechanism that contributes to the reprogramming of host tissue, thereby instigating alterations in tissue homeostasis and facilitating cancer progression [14]. Therefore, this review emphasizes the role of sEVs within EVs as key mediators of communication between tumor cells and CAFs. This section presents a concise review of EV characteristics, composition, and biogenesis, drawing on the latest research findings.

### 3.1. Characteristics and Composition of EVs

EVs are a highly heterogeneous class of particles secreted by all cell types [76]. To date, classification criteria for subpopulations of EVs have not been standardized. Based on the biogenesis pathway, EVs can be categorized into exosomes and microvesicles (also known as ectosomes). Exosomes are mainly dependent on MVBs for their release from the inside of the cell. Ectosomes or microvesicles are dependent on the plasma membrane released from the cell surface. Additionally, EVs can be classified according to the source of a particular cellular program, such as migrasomes generated by cell migration and apoptotic bodies generated by programmed cell death. Based on size, EVs are categorized into large EVs with diameters greater than 200 nm and small EVs with diameters less than 200 nm. Importantly, most of the current EV studies use ultracentrifugation to isolate EVs of different sizes rather than the biogenesis. Therefore, MISEV2023 recommended categorizing and defining different subpopulations of EVs based on their size. Notably, it also clarifies that sEVs include small ectosomes and exosomes [16]. Due to an insufficient understanding of the relationship between different EV subpopulations, there are no universally used molecular markers to isolate EV subpopulations. It is generally accepted that ectosomes are enriched for CD9 and CD81 on the cell surface, and exosomes are enriched for Alix, syntenin, CD9, and CD63 on the cell surface [77,78]. However, this strategy still has some limitations due to the specificity of different molecular markers and need to be further explored.

EVs contain diverse functional components, such as lipids, proteins, and nucleic acids [79]. Their bilayer membrane structure comprises various lipids, including sphingomyelin, phosphatidylinositol, ceramide, and cholesterol. These lipids contribute to the structural integrity of EVs and affect their secretion, release, cargo sorting, biogenesis, and various other functions [80]. Proteins in EVs can be categorized into non-specific and cell-specific proteins. Non-specific proteins include integrins, heat shock proteins (HSPs), members of the tetraspanin family (e.g., CD9, CD63, and CD81), components of the Endosomal Sorting Complex Required for Transport (ESCRT), such as Alix and the tumor susceptibility gene 101 (TSG101), and proteins involved in membrane transport, like RAB GTPases, annexins, and flotillins. Conversely, cell-specific proteins, including Major histocompatibility complex (MHC) classes I and II, vary according to the donor cell’s composition [81]. Furthermore, loading biologically active proteins into engineered EVs represents a promising therapeutic strategy in the foreseeable future [82].

The nucleic acid contents of EVs predominantly comprise DNA, mRNA, and various non-coding RNAs (ncRNAs). Among these, ncRNAs—including microRNAs (miRNAs), long non-coding RNAs (lncRNAs), circular RNAs (circRNAs), and PIWI-interacting RNAs (piRNAs)—are critical for modulating numerous biological processes. It is noteworthy that they are particularly influential in the malignant progression of tumors and have emerged as focal points in the research on early cancer diagnosis and prognosis [83]. However, Jeppesen, D. K. et al. recently proposed that the release of extracellular active DNA relies not on exosomes as carriers, but on a novel mechanism involving autophagy and MVB dependence. This insight offers a fresh perspective on evaluating EV components [84]. In addition, the database ExoCarta (http://www.exocarta.org) (accessed on 21 November 2024) provides access to multiple EV components identified from a wide range of organisms, such as 9769 proteins, 3408 mRNAs, and 2838 miRNAs, to date [85].

### 3.2. Biogenesis of EVs

Extracellular vesicles can be divided into two main subpopulations based on their biogenesis: ectosomes (microvesicles) and exosomes [86]. Ectosomes or microvesicles are released from the plasma membrane by outward budding. Unlike the biogenesis of ectosomes, exosomes first form early endosomes by the endocytosis of the plasma membrane. These early endosomes then encapsulate a variety of proteins and nucleic acids in the cytoplasm and mature into late endosomes, which form ILVs in the lumen of the organelle. The aggregation of ILVs transforms these endosomes into MVBs, a portion of which subsequently fuse with the plasma membrane and release ILVs to the extracellular medium as exosomes, while the rest of the MVBs undergo degradation within lysosomes [87,88].

The endosomal sorting complex required for transport (ESCRT)-mediated pathways or ESCRT-independent pathways is essentially required for the biogenesis of EVs [89]. ESCRT pathways mainly consist of ESCRT-0/I/II/III and VPS4 protein complexes. These complexes are involved in the sorting of ubiquitination products, the budding of ILVs, and membrane scission [90,91]. The ESCRT-independent pathway is dependent on lipids and lipid-associated proteins, such as the tetraspanins CD63, CD9, and CD81. These lipids, including ceramide, lysophospholipid and glycosphingolipid, mediate ILV biogenesis and sorting [92,93]. Unlike lipids and tetraspanins, the Rab GTPases Rab31 and Rab7 co-regulate ILV formation and exosome secretion in an antagonistic manner [94,95]. Additionally, a key study found that the syndecan/syntenin/ALIX complex pathway plays a pivotal role in EV biogenesis and release [96]. Although the biogenesis pathway of EVs is generally understood, additional mechanisms need to be further investigated to help unravel the functions performed by EVs.

## 4. Role of CAF-Derived sEVs in Tumor Progression

**Figure 2:**
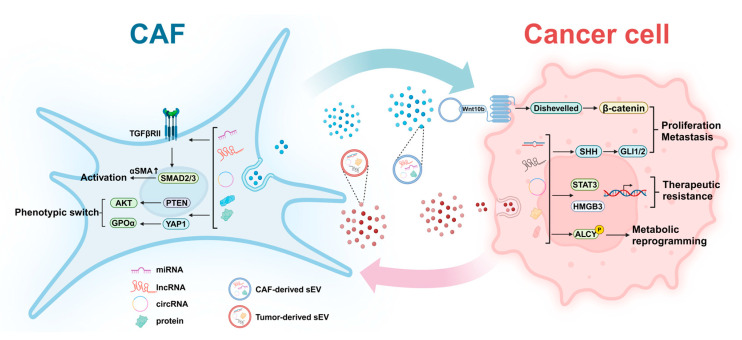
The sEV-mediated communication axis between CAFs and tumor cells. Tumor cells can interact with CAFs via sEVs to regulate tumor progression and stromal microenvironment formation.

The sEV-mediated communication axis between CAFs and tumor cells has a significant impact on cancer progression and the stromal microenvironment (Figure 2).

Numerous studies have affirmed the role of CAF-derived sEVs in cancer progression, including proliferation, metastasis, therapeutic resistance, and metabolism [21,97,98]. As such, the functions of sEVs secreted by CAFs are linked to their diverse molecular constituents, including non-coding RNAs, proteins, and other bioactive molecules, making them promising therapeutic targets for malignant tumors. This section explores the specific molecular mechanisms through which CAF-derived sEVs influence cancer progression and their effects on cancer cells (Table 1).

### 4.1. Proliferation and Metastasis

In malignant tumor populations, a key phenotypic characteristic is the deregulation of tumor cell proliferation [142]. Additionally, early preclinical studies have shown that metastasis—the process through which tumor cells spread to distant organs—entails the adaptation of these cells within unacquainted tissue microenvironments [143,144]. Proliferation and metastasis are critical to cancer progression, and sEVs derived from CAFs play a significant role in these two fundamental hallmarks of cancer [145]. In breast cancer, Luga and colleagues first confirmed that CAF-secreted sEVs with a high CD81 expression can promote breast cancer cell motility and metastasis via Wnt-planar cell polarity (PCP) signaling, and this response was dependent on the exosome component CD81 in CAFs [146]. Numerous studies have implicated that microRNAs in sEVs have significant functions in the interactions between CAFs and cancer cells, such as miR-500a-5p, miR-18b, miR-16 and miR138a [104,105,106]. In breast cancer, sEVs derived from CAFs that carry miR-500a-5p can enhance proliferation and metastasis by targeting ubiquitin-specific peptidase 28 (USP28) [105]. Moreover, exosomal miR-18b can promote breast cancer cell invasion and metastasis by specifically targeting 3′UTR of Transcription Elongation Factor A Like 7 (TCEAL7) [106]. Similar to miRNAs, certain long noncoding RNAs (lncRNAs) in sEVs derived from CAFs have been identified as having tumorigenic effects. For example, a recent study has shown that WEE2-AS1, highly expressed in CAF-derived sEVs, targeted MOB1A, accelerated MOB1A degradation, inhibited the Hippo pathway, and promoted cancer cell proliferation in colorectal cancer [109]. Additionally, exosomal LINC00659 can promote cancer cell proliferation, invasion, migration, and EMT progression by interacting directly with miR-342-3p and increasing Annexin A2 (ANXA2) expression in CRC cells [113]. In addition, the secretion of circular RNAs (circRNAs) in CAF-derived sEVs has been confirmed to have cancer-promoting abilities. For example, researchers verified that exosomal circ_0088300 derived from CAFs served as a sponge that directly targeted miR-1305, activated the JAK/STAT signaling pathway, and promoted proliferation, migration, and invasion in gastric cancer [126]. Similarly, in breast cancer, hypoxic CAF-secreted exosomal circHIF1A was shown to promote cell proliferation and display stem cell features by sponging miR-580-5p and regulating CD44 expression [102]. Recently, exosomal circTBPL1 derived from CAFs was discovered to facilitate breast cancer cell proliferation and metastasis by sponging miR-653-5p and downregulating its target gene, TPBG [108].

Finally, multiple proteins delivered by sEVs from CAFs also play an important role in the crosstalk between CAFs and cancer cells [147]. For example, in breast cancer, sEVs play an essential role in paracrine Wnt10b transport from CAFs to cancer cells. Importantly, p85α-deficient CAF-derived Wnt10b can significantly facilitate EMT induced by activating the Wnt signaling pathway [103]. As such, CAF-derived sEVs, which contained highly expressed Sonic Hedgehog (SHH), prominently improve the growth and migration of tumor cells via the Hedgehog signaling pathway in esophageal squamous cell carcinoma (ESCC) [135]. In pancreatic cancer, annexin A6 (ANXA6) was detected in high levels in CAF-derived sEVs. The exosomal transfer of ANXA6 was found to facilitate pancreatic cancer aggressiveness by the formation of the annexin A6/LDL receptor-related protein 1/thrombospondin 1 (ANXA6/LRP1/TSP1) complex [118]. Furthermore, autophagy-associated GPR64 was demonstrated to be enriched in hypoxic CAF-derived sEVs. The sEVs mediated the transfer of GPR64 from CAFs to cancer cells, which activated non-canonical NF-κB signaling to upregulate MMP9 and IL-8, improving the invasive abilities of the breast cancer cells [101]. CD9 on sEVs derived from CAFs plays a crucial role in the uptake of sEVs into pancreatic cancer cells and contributes significantly to pancreatic cancer progression [20].

Despite the extensive discussion in the existing literature regarding the promotion of cancer progression by CAF-derived sEVs, certain studies have discovered the secretion of anti-tumor sEVs secreted by CAFs, potentially signifying the existence of functionally heterogeneous populations of CAFs [148]. In a fibroblast-specific inducible focal adhesion kinase (FAK) knockout mice model, miR-16 and miR-148a enriched in FAK-null CAF-derived sEVs can significantly inhibit tumor cell activities and metastasis [104]. Moreover, LncRNA DACT3-AS1, found in sEVs derived from CAFs, has been verified to function as a suppressive regulator in malignant progression and enhance resistance to oxaliplatin by targeting the miR-181a-5p/sirtuin 1 (SIRT1) axis [127].

### 4.2. Therapeutic Resistance

The genomic and phenotypic heterogeneity inherent in malignant tumors renders certain cancers resistant to conventional adjuvant therapies [149,150]. Recent studies have revealed that the exosome-mediated transfer of ncRNAs can enhance resistance to various therapeutic approaches, including chemotherapy [151] and radiotherapy [115]. Targeting the transfer of exosomal ncRNAs may offer a novel strategy for managing therapeutic resistance in malignant tumors, potentially improving treatment outcomes and prognoses.

Cisplatin, which functions through interfering with DNA repair mechanisms, causing DNA damage and promoting cancer cells apoptosis, is a well-known chemotherapeutic drug [152]. Nonetheless, various types of cancer patients eventually develop cisplatin resistance and show a poor prognosis. A growing body of evidence indicates that many molecular mechanisms of cisplatin resistance are caused by CAF-derived sEVs. For instance, in hepatocellular carcinoma (HCC), circZFR was highly expressed in CAFs and CAF-derived sEVs. Thus, the latter can deliver circZFR to the recipient HCC cells, inhibit the STAT3/NF-κB signaling pathway, and facilitate HCC cells’ proliferation and chemoresistance to cisplatin [123]. In addition, miR-20a transported by CAF-derived sEVs can promote resistance to cisplatin by targeting PTEN and promoting PI3K/AKT signaling pathway activation in NSCLC [130]. Furthermore, in esophageal squamous cell carcinoma (ESCC), CAF-derived exosomal miR-21 and IL-6 synergistically promoted the generation of monocytic myeloid-derived suppressor cells (M-MDSCs) via activating STAT3 by IL-6 in an autocrine pathway. Meanwhile, researchers also demonstrated that M-MDSCs were associated with cisplatin resistance in patients with ESCC. Therefore, the combined inhibition of miR-21 and the blocking of IL-6 receptor may be a potential treatment for reversing cisplatin resistance in ESCC through the downregulation of STAT3 signaling [133]. Moreover, recent research has shown that sEVs secreted by CAFs, carrying LINC00355, upregulate CRKL expression by suppressing miR-34b-5p. This process consequently enhances cisplatin chemoresistance and accelerates EMT progression in CRC cells [117].

Other studies have suggested that the CAF-derived sEVs may promote resistance to other chemotherapy drugs of tumor cells as well, such as paclitaxel [138], gemcitabine [120], tamoxifen [107], and anti-pyrimidine drugs [110,131]. In ovarian cancer, the transfer of exosomal miR-21 secreted by neighboring CAFs in the omental tumor microenvironment suppresses cancer cell apoptosis and confers paclitaxel resistance via binding to its direct target APAF1 [138]. During gemcitabine treatment, CAF-derived sEVs may promote pancreatic cancer cells’ proliferation and resistance to gemcitabine. Importantly, in this study, researchers identified five CAF-derived exosomal miRNAs, including miR-21, miR-181a, miR-221, miR-222, and miR-92a, which confer gemcitabine resistance through targeting PTEN and downregulating the expression of PTEN [120]. Likewise, Qi et al. uncovered that exosomal miR-3171-5p derived from CAFs sponged ACSL4, leading to the inhibition of ferroptosis and the promotion of gemcitabine chemoresistance [121]. In breast cancer, CD63+ CAF-derived sEVs have been found to mediate cancer cell resistance to tamoxifen via exosomal miR-22 that binds its targets ERα and PTEN. Furthermore, this study also discovered that the packaging of miR-22 into sEVs secreted by CD63+ CAFs is mediated by SFRS1 [107]. Moreover, exosomal miR-146-5p secreted by CAFs was discovered to noticeably promote urothelial bladder cancer (UBC) cells’ stemness and chemoresistance. Mechanistically, miR-146-5p downregulated ARID1A and PRKAA2, which caused the activation of STAT3 and mTOR signaling [137]. Furthermore, a recent study discovered that exosome-packaged circBIRC6 from CAFs promote oxaliplatin resistance by mediating the SUMOylation of XRCC4 in PDAC, which may be a promising diagnostic and therapeutic target for PDAC [122].

Radiotherapy is widely used as a standard preoperative treatment thanks to its ability to reduce local recurrence and promote apoptosis of tumor cells [153]. Nonetheless, patients often have resistance to radiotherapy and poor prognoses, which is partially due to heterogeneous subpopulations of cancer cells and various components in the TME [154]. Of note, sEVs seem to play an important role in eliciting resistance to radiotherapy. Radiation-induced sEVs can irreparably deliver damaged DNA from cancer cells to the extracellular environment and inhibit apoptosis in cancer cells [155]. Expressly, the upregulated miR-93-5p in CAF-derived sEVs was confirmed to confer radioresistance in colorectal cancer cells via upregulating TGF-β3 and downregulating FOXA1 [115]. Similarly, they also found a high level of expression of miR-590-3p in CAF-derived sEVs compared with normal fibroblasts. The exosome-mediated transfer of miR-590-3p from CAFs to cancer cells improved resistance to radiotherapy abilities in colorectal cancer [114].

### 4.3. Metabolism

Cellular metabolism, a critical hallmark of cancer, plays a pivotal role in tumorigenesis. It enables the acquisition of essential nutrients from nutrient-deprived environments, supporting cell viability and the synthesis of new biomass [156]. SEVs from CAFs facilitate cancer progression by delivering a variety of metabolites to tumor cells, thereby enhancing cellular nutrition. For example, Zhao et al. demonstrated that sEVs secreted by CAFs could inhibit mitochondrial oxidative phosphorylation and increase glutamine-dependent reductive carboxylation and glycolysis, thereby strikingly reprogramming the metabolic machinery in cancer cells. Importantly, by using intra-exosomal metabolomics, researchers proved that CAF-derived sEVs contain intact metabolites, including amino acids, lipids, and tricarboxylic-acid cycle intermediates, which are utilized by cancer cells and promote tumor growth [139]. Moreover, in hormonal therapy-resistant (HTR) metastatic breast cancer, the full mitochondrial genome was identified in CAF-derived sEVs. The horizontal transfer of mitochondrial DNA (mtDNA) from CAF-derived sEVs, which acts as an oncogenic signal, can promote an exit from dormancy of therapy-induced cancer stem-like cells [99]. Moreover, a recent study described a CAF-derived exosomal lncRNA, known as TUG1, which promoted metastasis and glycolysis in liver cancer cells via the miR-524-5p/SIX1 axis [124]. Collectively, CAFs secrete sEVs packed with diverse proteins, non-coding RNAs, metabolites, and other bioactive molecules that are transported to tumor cells, promoting tumor architecture development, proliferation, metastasis, metabolism, therapeutic resistance, and immunosuppression. Strategically targeting these molecules and their intracellular pathways represents a promising approach for enhancing cancer diagnosis, treatment, and prognosis. Recently, there is growing evidence that focuses on treatment resistance and metabolic reprogramming in tumors. It is worth noting that CAF-derived sEVs play an important role in both. Therefore, in order to find key therapeutic targets, it is particularly important to delve into the detailed molecular mechanisms of tumor therapeutic resistance and metabolic reprogramming mediated by CAF-derived sEVs.

## 5. Role of Tumor-Derived sEVs in CAFs

As the main constituent of the TME, the crosstalk between cancer-associated fibroblasts and tumor cells plays a vital role in cancer progression. Accumulating evidence suggests that tumor-derived sEVs have a noteworthy impact on the development of the TME, promoting fibrosis, metastasis, immune response, and angiogenesis [157,158]. Specifically, sEVs derived from tumors have been shown to promote the activation of CAFs, including proliferation, invasion, differentiation, and phenotypic transformation of fibroblasts (Table 2) [18,159,160,161].

### 5.1. The Activation of CAFs

A plethora of proteins from tumor-derived sEVs has been identified and shown to play an important role in the activation of CAFs. For example, the TGF-β family of ligands is a primary signaling molecule for the activation of CAFs. In 2010, researchers determined that sEVs produced by cancer cells could trigger elevated α-SMA expression and the process of fibroblast differentiation into myofibroblasts. This process is highly correlated with the enrichment of TGF-β in cancer-derived sEVs [157]. Similarly, this result was demonstrated in gastric and bladder cancer models [10,171]. In addition, Wnt2B was significantly enriched in cervical cancer cell-derived sEVs and transferred into fibroblasts to promote the activation of fibroblasts into CAFs via the Wnt/β-catenin signaling pathway [174]. Furthermore, Li et al. found that survivin is highly expressed in breast cancer cells and released extracellularly in the form of sEVs, which upregulates SOD1 expression in normal fibroblasts and then converts them into CAFs [163]. In colorectal cancer, primary tumor secreted ITGBL1-loaded sEVs significantly promoted the activation of resident fibroblasts in remote organs by stimulating the TNFAIP3-mediated NF-κB signaling pathway [173]. Recently, Ma et al. uncovered that the tumor cell-derived gain-of-function (GOF) p53 protein could be wrapped in sEVs and be taken up by normal fibroblasts in the TME, which significantly promote fibroblasts’ conversion into CAFs and subsequently enhance cancer progression [165]. However, the function of proteins remains to be studied. These abovementioned results suggest that targeting sEV proteins and their signaling axis may be a potential therapeutic strategy for cancer.

Most ncRNA studies focused on tumor cells, but a growing body of evidence indicates their involvement in reprogramming normal fibroblasts into CAFs. Recent studies found that piwi-interacting RNAs (piRNAs) are expressed in human somatic tissues and play a pivotal role in the carcinogenesis of different types of tumors [18]. For instance, one such piRNA, piR-25783, was found to be enriched in ovarian cancer cell-derived sEVs. The transfer of exosomal piR-25783 from tumor cells to fibroblasts activated the TGF-β/SMAD2/SMAD3 signaling pathway in fibroblasts and promoted normal fibroblasts to differentiate into CAFs, along with the secretion of multiple cytokines, contributing to the progression of ovarian carcinoma [18]. Also, miR-9 was detected at high levels in tumor-derived sEVs. The exosome-induced transfer of miR-9 from cancer cells to normal fibroblasts participated in reprogramming normal fibroblasts into CAFs, thus contributing to tumor growth in breast cancer [162]. Similar to miRNAs and piRNAs, the secretion of lncRNAs from tumor cells has been proven to have the ability to activate CAFs. In ESCC, for example, lncRNA POU3F3 can be delivered from tumor cells to normal fibroblasts via sEVs. Upregulated lncRNA POU3F3 was involved in differentiation from normal fibroblasts to CAFs, and thus mediated cisplatin resistance in ESCC [179]. Moreover, in mouse models, triple-negative breast cancer cell-derived sEVs could promote the expression of multiple CAF markers and the activation of CAFs via the transfer of miR-125b [166]. Recently, He et al. demonstrated that exosomal circEHD2, regulated by heterogeneous nuclear ribonucleoprotein A2/B1 (hnRNPA2B1), was transferred to fibroblasts and converted fibroblasts to CAFs in renal cell carcinoma (RCC) [183]. Further mechanistic studies discovered that exosomal LINC00881 derived from osteosarcoma cells facilitated the conversion of normal lung fibroblasts into CAFs by sponging miR-29c-3p and regulating the expression of MMP2 [169]. Activated CAFs can significantly promote tumor progression by secreting cytokines, such as IL-6.

### 5.2. The Switch of Phenotype in CAFs

As previously mentioned, phenotypic heterogeneity in CAFs leads to functional diversity for cancer progression. The emerging understanding that CAFs have various tumor-promoting abilities but also potentially have tumor-restraining functions [184], which highlights the need to develop multiple phenotype-specific therapeutic strategies. There is increasing evidence demonstrating that tumor-derived sEVs can induce the switch of phenotype in CAFs, including proangiogenic and proinflammatory phenotypes [9,161,170,178]. For example, Fan et al. found that the transfer of miR-210 by lung cancer cell-derived sEVs may facilitate CAFs to develop a proangiogenic phenotype by modulating the JAK2/STAT3 signaling pathway. Importantly, ten-eleven translocation 2 (TET2) was defined as the target of miR-210 in CAFs, which participated in the proangiogenic phenotype switch of CAFs [161]. Furthermore, Zhou et al. found that HCC cells abundantly secrete sEVs that contain miR-21, which are transferred to HSCs and then promoted them to convert to CAFs and secrete angiogenic cytokines, including VEGF, MMP2, MMP9, and bFGF [9]. Moreover, researchers also described that miR-21 transferred by HCC cell-derived sEVs that directly targeted PTEN, leading to the activation of PDK1/AKT signaling in HSCs [9]. In addition to the proangiogenic phenotype, Fang et al. demonstrated that high-metastatic HCC cells secrete exosomal miR-1247-3p, which directly targets B4GALT3, leading to the activation of β1-integrin-NF-κB signaling in CAFs, and then activated CAFs boosted the development of tumor by secreting multiple proinflammatory cytokines, such as IL-6 and IL-8 [170]. This study also shows that the intercellular crosstalk between tumor cells and CAFs is mediated by sEVs that promote the lung metastasis of HCC [170]. Likewise, Mo et al. showed that ovarian cancer cells highly secreted an exosomal miRNA, hsa-miR-141-3p, and induced the switch of stromal fibroblasts into proinflammatory CAFs, promoting metastatic colonization by reducing the nuclear YAP1/TAZ ratio and facilitating stromal fibroblasts to produce GROα [178]. There is also evidence that sEV cargo miR-4736 suppressed autophagy by targeting ATG7 and enhanced CAFs activation in PDAC. An insightful discovery of this research is that intratumoral lnc-FSD2-31:1 may restrain the transfer of miR-4736 between CAFs and cancer cells, and more molecular mechanisms that may block the intracellular communication between CAFs and cancer cells need to be explored [19].

On the other hand, sEVs secreted by tumor cells can also inhibit the phenotypic switch of CAFs [180]. In head and neck squamous cell carcinoma (HNSCC), Wang and colleagues found that HPV + HNSCC cell-derived sEVs can significantly reduce the phenotypic transformation of CAFs. Specifically, miR-9-5p was found to be enriched in HPV + HNSCC cell-derived exosomes. After being absorbed by CAFs, miR-9-5p targeted NOX4 and downregulated the level of intracellular reactive oxygen species (ROS), which inhibited the TGFβ signaling-mediated phenotypic transformation of CAFs [180].

**Figure 3:**
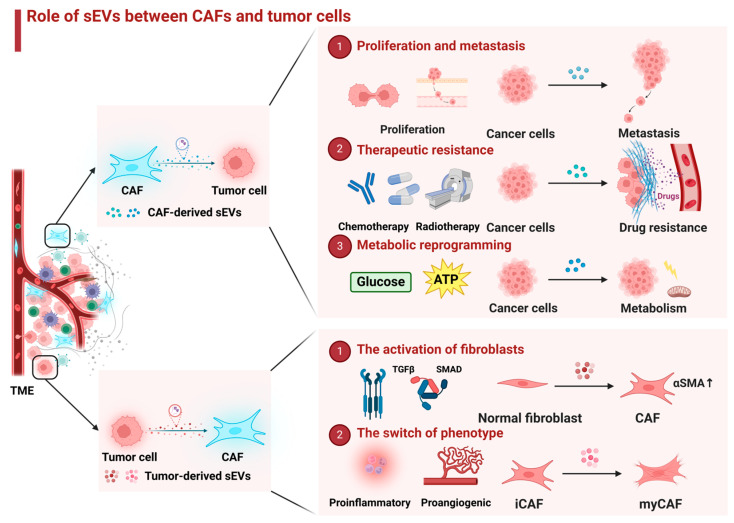
Role of sEVs between CAFs and tumor cells. Activated CAFs in the TME release sEVs to tumor cells and mediate both pro- and anti-tumor effects. The main effects include modulating tumor cell proliferation, metastasis, therapeutic resistance, and metabolic reprogramming. In addition, tumor cells also promote the activation of normal fibroblasts into CAFs and phenotypic switching of CAFs through sEVs.

Furthermore, cancer cell-derived sEVs can also exert their influence on CAFs through alternative pathways, such as promoting CAFs motility and reprogramming lipid metabolism [116,162]. Zhang et al. revealed that CRC cell-derived exosomal HSPC111 can modulate the lipid metabolism of CAFs by phosphorylating ATP-citrate lyase, leading to an upregulation in the level of acetyl-CoA [116]. Taken together, cancer cell-derived sEVs play a crucial role in CAF motility, invasion, activation, and phenotypical switch (Figure 3). Due to the heterogeneity of CAFs, different subpopulations may exert pro- or anti-cancer effects. For example, myCAFs can directly promote phenotypes such as proliferation and metastasis of tumor cells, iCAFs and apCAFs can mediate immunomodulation of tumor cells, but patients with adequate meCAFs respond better to immunotherapy. Therefore, in the future, in-depth studies on the phenotypic switching mechanism of CAFs, especially the conversion of pro-oncogenic CAFs, into anti-oncogenic CAFs, may serve as a potential clinical therapeutic strategy for cancer.

## 6. Clinical Application of Tumor- and CAF-Derived sEVs

In recent decades, the communication mediated by sEVs between tumor cells and CAFs has been extensively investigated for promoting tumor progression. Therefore, targeting sEVs derived from either tumors or CAFs can potentially serve as a promising strategy for cancer therapy. The compound GW4869 is widely recognized as the most frequently employed inhibitor of tumor-derived sEVs. However, recent mechanistic research has revealed that GW4869 may also impact exosome secretion in normal cells through an nSMase-mediated ESCRT-independent pathway, leading to undesirable in vivo side effects [185]. Hence, further studies on the mechanisms of exosome biogenesis and release from malignant tumor cells are warranted. Recently, the lineage kinase domain-like protein (MLKL) was demonstrated to exert endosomal compartment trafficking and the constitutive release of sEVs by the proteomic analysis of necroptotic extracellular vesicles [186]. In this regard, André-Grégoire, G et al. revealed that genetic, protein, and pharmacological interferences with MLKL significantly reduced exosome release from glioblastoma stem cells (GSCs) and suppressed the progression of patient-derived GSCs [187]. In addition, the activation of Vitamin D receptor (VDR) signaling in CAFs can inhibit the secretion of CAF-derived miR-10a-5p, thereby effectively suppressing the progression of PDAC [188,189]. It is suggested that, in addition to directly inhibiting the secretion of sEVs from tumor cells or CAF cells of cellular origin, targeting the inhibition of oncogenic molecule release from these sEVs could also be considered as a potential therapeutic strategy. Furthermore, a variety of small-molecule drugs have been loaded into engineered sEVs as a therapeutic payload to tumors, such as paclitaxel [190,191], gemcitabine [192], small interfering RNA (siRNA) [193], and CRISPR/Cas9 [194]. Recently, a pivotal study indicated that miR-876-3p antogomir, packaged and delivered by sEVs derived from CAFs, can target GATA-binding protein 1 (GATA1) and reduce cisplatin resistance in Oral squamous cell carcinoma (OSCC) cells. This finding highlights a notable strategy where oncomiR antogomirs are encapsulated within sEVs for tumor therapy [195]. However, additional small-molecular drugs and inhibitors that can be effectively incorporated into sEVs need to be explored, and how to elicit these strategies into high-efficiency and low-toxicity clinical translations needs to be discussed.

**Figure 4:**
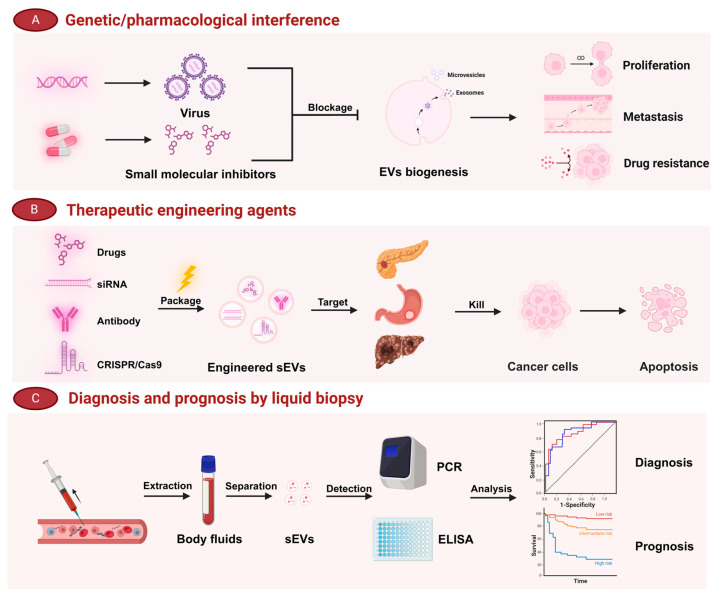
Clinical application related to sEV biogenesis and function. Several approaches have been designed to specifically block the biogenesis of sEVs in tumor cells, mainly including the use of viruses carrying functional nucleic acids or small-molecule inhibitors. Moreover, engineering sEVs to carry drugs, siRNA, antibodies, or CRISPR/Cas9 can directly target the lesion and inhibit tumor cells. In addition, liquid biopsy analysis of sEVs is beneficial for diagnosis and prognosis.

A growing body of evidence has demonstrated that sEVs are more sensitive and specific than CTCs and ctDNA in liquid biopsy for cancer early diagnosis and prognosis prediction [196]. The combined detection of exosomal cargo and classical biomarkers, such as CEA, CA125, and CA199, holds great potential as a prospective diagnostic and prognostic strategy in the future. Recently, exosomal miR-30c showed a high predictive value with an area under the curve of 87.2% in NSCLC patients. In this regard, researchers suggested that low exosomal miR-30c and the existence of autophagic-activated CTCs may serve as independent predictive biomarkers for NSCLC prognosis [197]. Moreover, Brokāne et al. discovered two novel exosome-enclosed RNA biomarkers—GLO1 and NKX3-1—with AUCs of 0.85 and 0.71 in the test and validation cohorts for prostate cancer (PCa), respectively. Notably, researchers highlighted that the combination of GLO1, NKX3-1, and PSA has a pleasantly complementary nature in liquid biopsies of Pca [198]. Although a number of sEV-based liquid biopsies in clinical applications of cancer diagnosis and prognosis has been verified [199,200,201], more sensitive and specific biomarkers still urgently need to be explored in larger clinical samples and trials (Figure 4).

## 7. Perspectives and Challenges

Recent research has increasingly focused on the TME as a potential source for novel cancer therapies. The TME, comprising diverse cell types within the ECM—such as immune cells, endothelial cells, and CAFs—engages in complex interactions with cancer cells during tumor progression. CAFs, a prominent and heterogeneous cell type within the TME, exert their influence through the remodeling of vasculature and the ECM and the secretion of soluble factors and sEVs [2]. Recent advancements have been made in understanding the role of sEVs in facilitating communication between cancer cells and CAFs, as well as other stromal cells. On the one hand, cancer cells can deliver multiple bioactive molecules to CAFs and influence the activation of CAFs via sEVs [165,166]. On the other hand, sEVs secreted by CAFs can also regulate cancer cells growth, metastasis, invasion, immune evasion, drug resistance, metabolic reprogramming, etc. [21,97,202]. Furthermore, it is well known that activated CAFs promote tissue fibrosis and ECM stiffening. Notably, a pivotal study found that a stiff ECM promotes the activation of Rab 8 by Akt, which promotes sEVs secretion in the HCC cell line. These sEVs secreted by stiff ECM-promoted tumor cells can significantly facilitate the activation of the Notch pathway and tumor growth in mouse xenograft models [203]. It is suggested that tumor-CAF-ECMs may form a sustained pro-tumor positive feedback loop via sEVs, namely sEVs from tumor cells promote the activation of CAFs, activated CAFs secrete more collagen and stiffen the ECM, and a stiff ECM further promotes tumor cells to secrete pro-carcinogenic or pro-CAF-activated sEVs. Despite the recent progress, how sEV cargo from CAFs or other stromal cells in the TME has an influence on cancer progression remains largely unknown. In addition, the direct effect of CAF-derived sEVs on immune cells or other stromal cells in the TME remains to be elucidated, which is a very promising area for future research. Moreover, the investigation of additional cargo from cancer cell-derived sEVs, which potentially mediate a variety of functions in CAFs, is necessary.

At present, the precise impact mediated by sEVs derived from different cell populations in the TME remains unexplored. Importantly, the in-depth study of the interaction between TME cells and cancer cells is of great clinical relevance. First, sEVs have been substantiated as enduringly present in bodily fluids, exhibiting stability, and presenting a broad array of information that aptly reflects the dynamic state of tumor progression. The cargo of sEVs can serve as a potential biomarker in liquid biopsy for cancer diagnosis and prognosis prediction. Although, on account of the high heterogeneous characteristics of sEVs, the acquisition of their molecular mechanisms has posed great technical challenges [204,205]. Additionally, engineered sEVs with surface modifications have been improved as a possible therapeutic strategy [206]. For example, neutrophile-derived sEVs (N-Ex) can transfer cytotoxic proteins, activate the caspase signaling pathway, and induce tumor cell apoptosis. It is noteworthy that, after decorating with superparamagnetic iron oxide nanoparticles (SPIONs), N-Ex has shown a greater tumor-targeting therapeutic effect [207]. As more mechanisms of the interaction between CAFs or other stromal cells in the TME and tumor cells are discovered, further insights into cancer therapy will likely be gained.

## 8. Conclusions

In this review, we discussed and summarized the latest research on the crosstalk between CAFs and tumors via sEVs that promote tumor malignant progression. We further highlight the clinical therapeutic applications based on CAF-derived and tumor-derived sEVs, thus providing new potential strategies for inhibiting tumor malignant progression and overcoming therapeutic resistance. Due to the critical role of CAFs and tumor intercommunication, future studies on the function and mechanism of sEVs are needed to develop novel clinical therapeutic strategies for cancer.

## Figures and Tables

**Table 1:** Contents in CAF-derived sEVs.

Cancer Type	Model and Specie	Molecule	Mechanism	Effect	Ref.
Breast cancer	Human CAFs and xenograft mouse models	mtDNA	Promote and escape from metabolic quiescence	Increase metabolism and promote endocrine therapy resistance	[99]
	Human fibroblasts and orthotopic xenograft mouse models	CD81	Mobilize autocrine Wnt-PCP signaling to drive invasive behavior of cancer cells	Promote metastasis, migration, and invasion	[100]
	Human CAFs and in vitro models	GPR64	Stimulate non-canonical NF-κB signaling to upregulate MMP9 and IL-8 in breast cancer cells	Promote cancer cell invasion	[101]
	Human CAFs and in vitro models	CircHIF1A	Sponge miR-580-5p by regulating CD44 expression in cancer stem cells	Promote the stemness of cancer stem cells	[102]
	Human CAFs and in vitro models	Wnt10b	Promote EMT by the canonical Wnt pathway	Promote metastasis	[103]
	Mouse CAFs and cKO mouse models	miR-16 and miR-138a	Enrich exosomes from FAK-null CAFs and reduce tumor cell activities	Inhibit breast cancer cell migration and metastasis	[104]
	Human CAFs and xenograft mouse models	miR-500a-5p	Target USP28 and downregulate USP28	Promote proliferation and metastasis	[105]
	Human CAFs and xenograft mouse models	miR-18b	Promote nuclear Snail ectopic by targeting TCEAL7 to activate the NF-κB pathway	Promote cancer invasion and metastasis	[106]
	Human CD63^+^ CAFs and xenograft mouse models	miR-22	Downregulate ERα and PTEN expression	Promote tamoxifen resistance	[107]
	Human CAFs and xenograft mouse models	CircTBPL-1	Sponge miR-653-5p and stabilize TPBG	Promote breast cancer proliferation, migration, and invasion	[108]
Colorectal cancer	Human CAFs and xenograft mouse models	WEE2-AS1	Promote degradation of MOB1A and inhibit the Hippo pathway	Promote growth of CRC cells	[109]
	Human CAFs and xenograft mouse models	miR-181d-5p	Target NCALD to inhibit the 5-FU sensitivity of CRC cells	Promote resistance to 5-FU	[110]
	Human CAFs and xenograft mouse models	miR-200b-3p	Target HMBG3 and suppress HMBG3	Promote resistance to 5-FU	[111]
	Human CAFs and in vitro models	miR-625-3p	Inhibit the CELF2/WWOX pathway	Promote migration, invasion, EMT, and chemoresistance	[112]
	Human CAFs, NFs, and in vitro models	LINC00659	Interact directly with miR-342-3p to increase ANXA2 expression in cancer cells	Promote proliferation, invasion, and migration	[113]
	Human CAFs and xenograft mouse models	miR-590-3p	Target CLCA4 and downregulate CLCA4	Promote radioresistance	[114]
	Human CAFs, NFs, and xenograft mouse models	miR-93-5p	Downregulate FIXA1 and upregulate TFGB3	Promote redioresistance	[115]
	Human CAFs and xenograft mouse models	HSPC111	Phosphorylate ATP-citrate lyase (ACLY) and upregulate the level of acetyl-CoA	Reprogram lipid metabolism in CAFs	[116]
	Human CAFs, NFs, and in vitro models	LINC00355	Upregulate CRKL via inhibiting the expression of miR-34b-5p	Promote EMT and chemoresistance	[117]
Pancreatic cancer	Human CAFs and orthotopic xenograft mouse models	ANXA6	Increase PDA aggressiveness by uptake of the ANXA6/LRP1/TSP1 complex	Promote growth and metastasis of PDAC	[118]
	Human ANXA6^+^ CAFs and xenograft mouse model	CD9	Mediate the uptake of ANXA6^+^ exosomes from CAFs into PDAC cells	Promote MAPK pathway activity, cell migration, and EMT	[20]
	Human CAFs	Snail	Increase chemoresistance-inducing factor in epithelial cells	Promote proliferation and gemcitabine chemoresistance	[119]
	Human CAFs and xenograft mouse models	miR-21, miR-181a, miR-221, miR-222, and miR-92a	Target PTEN and suppress PTEN expression	Promote proliferation and gemcitabine chemoresistance	[120]
	Human CAFs, NFs, and xenograft mouse models	miR-3173-5p	Sponge ACSL4 and inhibit ferroptosis	Promote gemcitabine chemoresistance	[121]
	Human CAFs, organoids in vitro, and xenograft mouse model	circBIRC6	Bind with XRCC4 and facilitate XRCC4 chromatin localization via SUMOylation modulation	Promote oxaliplatin chemoresistance	[122]
Liver cancer	Human CAFs and xenograft mouse models	circZFR	Inhibit the STAT3/NF-κB pathway	Promote cisplatin chemoresistance	[123]
	Human CAFs and xenograft mouse models	TUG1	Regulate the miR-524-5p/SIX1 axis	Promote cancer cell migration, invasion, and glycolysis	[124]
	Human CAFs, NFs, and in vitro models	miR-150-3p	Not mentioned	Suppress migration and invasion	[125]
Gastric cancer	Human CAFs, NFs, and in vitro models	Circ_0088300	Sponge and target miR-1305	Promote proliferation, migration, and invasion	[126]
	Human CAFs and xenograft mouse models	DACT3-AS1	Target miR-181a-5p/SIRT1 axis	Suppress malignant transformation and oxaliplatin resistance	[127]
	Human CAFs and xenograft mouse models	HSF1	Upregulate INHBA and THBS2	Promote aggressive gastric cancer phenotypes	[128]
Lung cancer	Human CAFs and xenograft mouse model	MEG3	Sponge miR-15a-5p and upregulate CCNE1 expression	Promote cisplatin chemoresistance	[129]
	Human CAFs, NAFs, and xenograft mouse models	miR-20a	Target PTEN and enhance PI3K/AKT pathway activation	Promote cancer cell proliferation and cisplatin chemoresistance	[130]
Lymphoma	Human CAFs and xenograft mouse models	miR-4717-5p	Suppress the expression of ENT2 and induce anti-pyrimidine drug resistance	Promote anti-pyrimidine drug resistance	[131]
ESCC	Human CAFs and xenograft mouse models	RIG-I/IFN-β	Affect chemosensitivity to cisplatin via RIG-I/IFN-β signaling	Promote proliferation and cisplatin resistance	[132]
	Human CAFs and in vitro models	miR-21	Activate STAT3 and promote the generation of M-MDSCs	Promote cisplatin resistance	[133]
	Human CAFs, NFs, and xenograft mouse models	miR-3656	Target ACAP2 and downregulate ACAP2	Promote proliferation, migration, and invasion	[134]
	Human CAFs and xenograft mouse models	Sonic Hedgehog	Activate the Hedgehog signaling pathway	Promote the growth and progression of ESCC	[135]
OSCC	Human CAFs and xenograft mouse models	Lysyl oxidase (LOX)	Interact with periostin, fibronectin, and bone morphogenetic protein-1, and activate the FAK/paxillin/YAP pathway	Promote collagen crosslinking and EMT	[136]
Bladder cancer	Human CAFs and in vitro models	miR-146-5p	Target and downregulate PRKAA2 and 3’UTR of mRNAs of ARID1A	Promote stemness and chemoresistance	[137]
Ovarian cancer	Human CAFs, CAAs, and xenograft mouse model	miR-21	Target APAF1 and suppress APAF1 expression	Promote paclitaxel resistance	[138]
Prostate cancer	Human CAFs and in vitro models	metabolites	Inhibit mitochondrial oxidative phosphorylation	Increase glucose metabolism	[139]
	Human CAFs, NFs, and xenograft mouse models	miR-1290	Target GSK3β and inhibit GSK3β/β-catenin signaling	Promote cancer cell growth and metastasis	[140]
Renal cell carcinoma	Human CAFs and xenograft mouse models	miR-181d-5p	Suppress the expression of RNF43 and activate the Wnt/β-catenin signaling pathway	Promote cancer stemness and tumor progression	[141]

**Table 2:** Contents of tumor-derived sEVs.

Cancer Type	Specie and Model	Molecular	Mechanism	Effect	Ref.
Breast cancer	Human NFs and xenograft mouse models	miR-9	Modulate genes mainly involved in cell motility and extracellular matrix remodeling pathways	Enhance cell motility and the switch to CAF phenotype	[162]
	HFF-1 cells and xenograft mouse models	Survivin	Target SOD1 and upregulate SOD1	Induce the differentiation of fibroblasts to myofibroblasts	[163]
	Human NFs and in vitro models	miR-146a	Downregulate TXNIP and activate the Wnt signaling pathway	Promote the activation of CAFs	[164]
	Human NFs and xenograft mouse models	GOF P53	Selectively combine with HSP90	Promote the activation of CAFs	[165]
	Human fetal fibroblasts and xenograft mouse models	miR-125b	Not mentioned	Promote the activation of CAFs	[166]
Lung cancer	HLF-1 cells and in vitro models	α-SMA	Not mentioned	Promote cell proliferation and inhibit cell apoptosis in normal lung fibroblasts	[167]
	Human lung fibroblasts and xenograft mouse models	miR-3473b	Activate NF-κB signaling	Induce pre-metastatic niche formation	[168]
	NIH/3T3 cells and xenograft mouse models	miR-210	Activate the JAK2/STAT3 signaling pathway	Induce the switch of CAFs to the proangiogenic phenotype	[161]
	HFL-1 cells and in vitro models	LINC00881	Upregulate MMP2 and promote the secretion of pro-inflammatory cytokines	Induce the activation of lung fibroblasts	[169]
Liver cancer	MRC5 cells and xenograft mouse models	miR-1247-3p	Target B4GALT3 and activate β1-integrin-NF-κB signaling	Activate CAFs and induce the switch of CAFs to the proinflammatory phenotype	[170]
	Human HSCs and in vitro models	miR-21	Target PTEN and activate the PDK1/AKT signaling pathway	Induce the switch of CAFs to the proangiogenic phenotype	[9]
Pancreatic cancer	Human CAFs and orthotopic xenograft mouse model	miR-4736	Suppresses autophagy and target ATG7	Induce the activation of CAFs	[19]
Gastric cancer	HucMSCs and in vitro models	TGFβ	Activate the SMAD signaling pathway	Induce the differentiation of hucMSCs to CAFs	[171]
	Human NFs and in vitro models	miR-27a	Target CSRP2 and downregulate CSRP2	Induce the differentiation of fibroblasts to CAFs	[172]
Colorectal cancer	CCD-18Co, WI-38, and xenograft mouse models	miR-1249-5p, miR-6737-5p, and miR-6819-5p	Target TP53 and downregulate TP53	Promote cell proliferation in CAFs	[159]
	WI-38, LX-2 and xenograft metastatic tumor mouse models	ITGBL1	Activate the TNFAIP3-mediated NF-κB signaling pathway	Induce the activation of CAFs	[173]
Cervical cancer	Human NFs and xenograft mouse models	Wnt2B	Activate the Wnt/β-catenin signaling pathway	Induce the differentiation of fibroblasts to CAFs	[174]
Bladder cancer	Human NFs and in vitro models	TGFβ	Activate the SMAD signaling pathway	Induce the differentiation of fibroblasts to CAFs	[10]
Melanoma cancer	NIH/3T3 cells and graft mouse models	miR-155-5p	Target SOCS1 and activate the JAK2/STAT3 signaling pathway	Induce the switch of CAFs to the proangiogenic phenotype	[175]
	NIH/3T3 cells and in vitro models	Gm26809	Not mentioned	Induce the differentiation of fibroblasts to CAFs	[176]
	MEF cells and in vitro models	miR-21	Downregulate TIMP3 and upregulate MMP	Promote the invasion of CAFs	[160]
Ovarian cancer	Human CAFs and in vitro models	miR-124	Target miR-124 and decrease α-SMA and FAP expression	Induce the differentiation of fibroblasts to CAFs	[177]
	Human omental fibroblasts and metastatic tumor mouse model	piR-25783	Activate the TGFβ/SMAD2/SMAD3 signaling pathway	Induce the differentiation of fibroblasts to myofibroblasts	[18]
	Human stromal fibroblast and cKO mouse model	miR-141	Target YAP1, reduce the nuclear YAP1/TAZ ratio, and facilitate stromal fibroblasts to produce GROα	Reprogram stromal fibroblasts into proinflammatory CAFs	[178]
ESCC	Human CAFs, NFs, and in vitro models	POU3F3	Not mentioned	Induce the differentiation of fibroblasts to CAFs	[179]
HNSCC	HGF-1 cells and in vitro models	miR-9-5p	Downregulate NOX4 and inhibit TGFβ signaling	Inhibit fibroblast phenotypic transformation	[180]
	Human NFs and in vitro models	miR-192/215	Target Caveolin-1 (CAV1) and promote the TGFβ signaling pathway	Promote CAF-like differentiation of the fibroblasts	[181]
	Human CAFs, NFs, and xenograft mouse model	TGFβ1	Regulate fibronectin	Promote the reprogramming of normal fibroblasts into CAFs	[182]
RCC	MRC5 cells and xenograft mouse model	circEHD2	Not mentioned	Induce the differentiation of fibroblasts to CAFs	[183]

## Abbreviations

CAFs	Cancer-associated fibroblasts
sEVs	Small extracellular vesicles
TME	Tumor microenvironment
MSCs	Mesenchymal stem cells
CSCs	Cancer stem cells
ECM	Extracellular matrix
CRC	Colorectal cancer
BCCs	Basal cell carcinomas
HSCs	Hepatic stellate cells
HCC	Hepatocellular carcinoma
EMT	Epithelial–mesenchymal transition
IMC	Imaging mass cytometry
NSCLC	Non-small-cell lung cancer
FAP	Fibroblast activation protein
FSP1	Fibroblast-specific protein 1
S100A4	S100 calcium-binding protein A4
MR	Mechanoresponsive
IM	Immunomodulatory
PDAC	Pancreatic ductal adenocarcinoma
myCAFs	Myofibroblastic CAFs
iCAFs	Inflammatory CAFs
apCAFs	Antigen-presenting CAFs
HGF	Hepatocyte growth factor
FGF7	Fibroblast growth factor 7
ILVs	Intraluminal vesicles
MVBs	Multivesicular bodies
HSPs	Heat shock proteins
ESCRT	Endosomal Sorting Complex Required for Transport
MHC	Major histocompatibility complex
ncRNAs	Non-coding RNAs
miRNAs	MicroRNAs
lncRNAs	Long non-coding RNAs
circRNAs	Circular RNAs
piRNAs	PIWI-interacting RNAs
PCP	Planar cell polarity
USP28	Ubiquitin-specific peptidase 28
TCEAL7	Transcription elongation factor A like 7
ANXA2	Annexin A2
SHH	Sonic Hedgehog
ANXA6	Annexin A6
FAK	Focal adhesion kinase
SIRT1	Sirtuin 1
M-MDSCs	Myeloid-derived suppressor cells
UBC	Urothelial bladder cancer
HTR	Hormonal therapy-resistant
mtDNA	Mitochondrial DNA
hnRNPA2B1	Heterogeneous nuclear ribonucleoprotein A2/B1
RCC	Renal cell carcinoma
TET2	Ten-eleven translocation 2
HNSCC	Head and neck squamous cell carcinoma
ROS	Reactive oxygen species
GSCs	Glioblastoma stem cells
VDR	Vitamin D receptor
siRNA	Small interfering RNA
GATA1	GATA-binding protein 1
OSCC	Oral squamous cell carcinoma
PCa	Prostate cancer
N-Ex	Neutrophil-derived exosome
SPIONs	Superparamagnetic iron oxide nanoparticles
